# Outbreak of scurvy in Tana River County, Kenya: A case report

**DOI:** 10.4102/phcfm.v10i1.1811

**Published:** 2018-10-25

**Authors:** Peter Halestrap, Sue Scheenstra

**Affiliations:** 1 Family Medicine Department, Africa Inland Church, Kijabe Hospital, Kenya; 2 Africa Inland Church Health Ministries, Kijabe Hospital, Kenya

## Abstract

Over a five-month period, clinicians in Tana River County Kenya observed an increasing number of patients presenting to health facilities with a common collection of symptoms including fevers, joint pains and gum hypertrophy. After initial investigative and management strategies failed to reveal a diagnosis, patients were empirically commenced on ascorbic acid for presumed scurvy. This strategy resulted in the rapid resolution of symptoms in 65 patients within two weeks.

## Introduction

### Background

In 2016–2017 East Africa experienced the worst drought to hit the region for 60 years, with the government of Kenya and international organisations declaring it a national emergency.^1^ The impact on food supplies in the region resulted in the re-emergence of dietary associated conditions such as scurvy. Many clinicians have never seen an active case of scurvy and therefore may not consider it as part of their differential diagnosis

### Case report

In December 2016 physicians at Africa Inland Church (AIC) Kijabe Hospital were contacted about an outbreak of a ‘mystery disease’ affecting people in Tana River County, Kenya. The first case had occurred in August and clinicians in the region were seeing increasing number of patients with similar symptoms.

**FIGURE 1:**
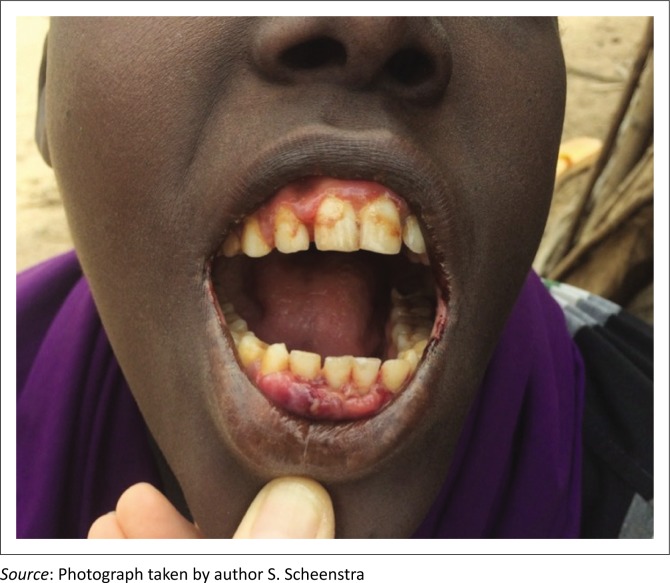
Gum Hypertrophy.

Patients were presenting with low-grade fevers, lethargy and pains in the muscles and joints. An inability to completely extend the knees was a common clinical finding with most patients walking with a slow hunched gait, and the most severely affected being bedbound. Another common complaint was that of gum hypertrophy and bleeding which could affect the ability of patients to eat (see Figure 1).

Examination of the patients revealed low-grade fevers (37.5 °C – 38 °C) and tenderness at the back of the legs, particularly behind the knees. There was joint stiffness that limited a full range of movement with significant distress. Some of the patients displayed active joint synovitis with associated swelling. Patients tended to have overt gum hypertrophy, though petechiae or perifollicular hyperkeratotic papules were not noted. Cardiovascular examination and respiratory examinations were normal and there was no evidence of hepatosplenomegally.

The condition was affecting both sexes equally and all age groups. The condition seemed to have a higher prevalence in those with long-term disabilities or other factors associated with lower socio-economic status. At the time of referral over 30 patients with this collection of symptoms had been reviewed in primary health care centres. Of importance, the region had experienced a recent period of drought and many of the local community blamed the outbreak of the disease on a lack of milk, a staple of the diet.

The remote location of these cases restricted the number of investigations that could be performed. One patient presenting early in the outbreak had been referred to a tertiary hospital for a biopsy of the gum lesion to exclude malignancy. The histology from this sample showed inflammatory changes only.

The low-grade fevers led to an initial presumption that the outbreak was infective in origin. Brucellosis was considered, given the presence of joint pains, and several patients were placed on empirical antibiotics but did not show any improvement. Arboviruses such as Chikungunya were also considered although due to the remote setting, it was not possible to test for these.

Reviewing the case, clinicians at AIC Kijabe Hospital thought that the symptoms may represent scurvy and recommended empirical treatment with ascorbic acid. Patients were placed on 100 mg ascorbic acid four times a day for two weeks. Those who were still symptomatic following this course of therapy were continued on the same dose of ascorbic acid for a further two weeks.

**FIGURE 2:**
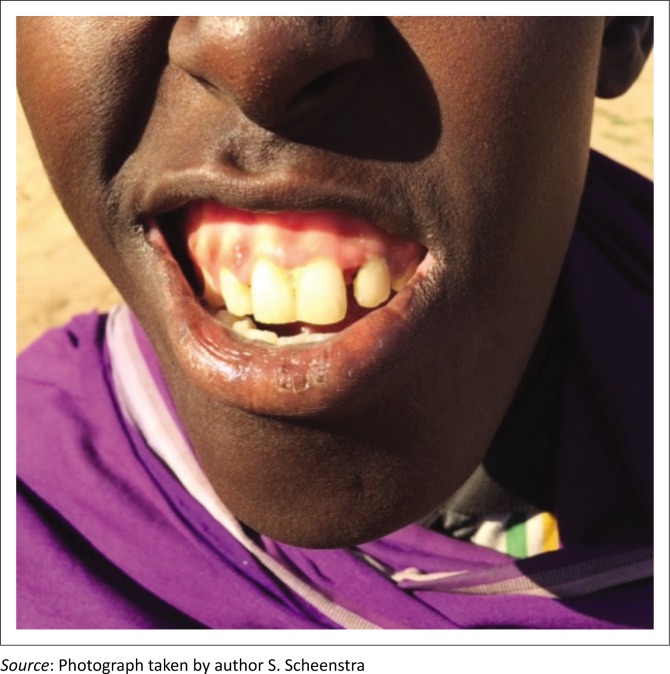
Resolution of gum hypertrophy following two weeks of ascorbic acid therapy.

Over the next two weeks 65 people were treated for scurvy with rapid resolution of their clinical symptoms and signs (Figure 2 shows the same patient as Figure 1 after two weeks ascorbic acid therapy). Patients were initially followed up by phone, as the region is remote and a sparsely populated region. After two weeks, a team visited the region in person to document the progress of the cases.

Local government officials and neighbouring clinics were informed about the outbreak so that other clinicians seeing similar cases would be able to instigate appropriate therapy. Alongside the administration of ascorbic acid, clinicians in Tana River County highlighted the importance of vitamin C in the diet and helped identify available sources in the community.

## Discussion

Scurvy is one of the oldest diseases known to mankind and as early as the 11th century sailors were advised to take with them a supply of fresh fruit and vegetables to ward off this condition.^2^ The disease spectrum is varied and includes dental, dermatological, bone and systemic manifestations. Common symptoms in adults include: gum disease and tooth loosening, weakness and irritability and pains in the muscles and joints of the legs. On examination many patients will display follicular hyperkeratosis, swollen joints, swollen bleeding gums and peripheral oedema.^3^ The preference to maintain the knees and hips in flexion, as seen in many of our cases, is known as the ‘pithed-frog’ position and is estimated to be present in half of infant patients.^4^ Untreated scurvy may lead to death in all age groups.

The diagnosis of scurvy can be made when there are clinical manifestations consistent with vitamin C deficiency, alongside a history of inadequate dietary intake. Biochemical indices including vitamin C levels in the blood and classical radiological changes can confirm the diagnosis. The most common change to be seen on plain radiographs is osteopenia, although subperiosteal elevation, a ground-glass appearance of the bone cortex and alveolar bone reabsorption may also be seen.^4^ In practice however, as in this case, often the best way to confirm the diagnosis is to watch for the resolution of symptoms with vitamin C treatment, since this therapy is cheap and has few side effects.

Sporadic cases of scurvy continue to be seen in the elderly, children with mental or physical disabilities and those with abnormal dietary habits throughout the world. Recent studies ^5^ have also suggested that subclinical deficiency in ascorbic acid may be the cause of poor wound healing in some patients even in the absence of the classical hallmark symptoms. Larger outbreaks of scurvy, such as the one recorded here, have rarely been documented since the late 1940s. The last noted outbreak in Kenya was in 1994, though it is possible that smaller outbreaks have gone undocumented. In the late 1980s high levels of scurvy were documented in refugee camps in Somalia and Sudan. These were due to the low levels of vitamin C in the relief food being supplied ^6^ and resulted in recommendations by the World Health Organization (WHO) for vitamin C to be added to relief food at an early stage.

This outbreak came in the midst of a long drought and the resultant dietary changes were the likely precipitant. Consistent with this hypothesis is the fact that the first people to present with symptoms were lactating mothers, people with disabilities and the poor, those persons in the demographics who often struggle when dietary resources are scarce.

The Orma people are semi-nomadic pastoralists living in Tana River County, a semi-arid desert region of Kenya. Many live deep in the bush and do not eat cultivated citrus fruits, mangoes or many of the vegetables which are rich in vitamin C. They, therefore, may not meet the World Health Organization’s recommended daily requirement of vitamin C for an adult. This 40 mg per day dose can be met by one orange, large tomato or helping of green vegetables. When the rains and pastures are good, the Orma drink abundant raw cow’s milk as the staple of their diet. When consumed raw cow’s milk may provide a dose of 6.5 mg – 10 mg vitamin C, a dose which has been found to be sufficient to prevent scurvy.^7^ When milk levels are low, such as in periods of drought, the milk tends to be utilised by the Orma to create Chai, a hot tea beverage. The boiling of the milk in this way will lower the vitamin C content and may be a contributing factor to the development of scurvy. This is consistent with the observation that the increase in scurvy in the later part of the 19th century coincided with an increased use of heat treated milk.^8^

## Ethical consideration

Informed written consent was obtained from the patient photographed. No other patient identifiable factors are contained within the manuscript.

## Conclusion

Scurvy is often considered a historical ailment; however, here we document an outbreak of the disease in a semi-arid region of Kenya, likely precipitated by a period of drought. The lack of clinician familiarity with scurvy likely led to delayed diagnosis and unnecessary investigations. Rapid clinical improvement and, therefore, confirmation of the diagnosis was seen with the empirical use of ascorbic acid. The famine that resulted in this scurvy outbreak also put patients at risk of other disease associated with malnutrition, and clinicians would be wise to remember other ‘historical’ elements associated with vitamin deficiencies such as beriberi and pellagra in their differentials.

### Teaching points

- The importance of clinicians remembering ‘historical’ ailments such as scurvy.
- Taking a thorough social history enables clinicians to identify possible environmental risk factors for diseases and work with communities to find preventative strategies for dietary diseases.
- The administration of ascorbic acid effectively results in large health gains at minimal cost.
